# Prospective evaluation of an anti-cancer drugs management programme in a dedicated oral therapy center (DICTO programme)

**DOI:** 10.1007/s12032-020-01393-7

**Published:** 2020-07-25

**Authors:** Elise Deluche, Tiffany Darbas, Kevin Bourcier, Loic Montangon, Geraldine Bayard, Evelyne Caille, Julie Querrioux, Chantal Suchaud, Sonia Zabaleta, Sabine Chaput, Valerie Le Brun-Ly, Julia Pestre, Laurence Venat, Frédéric Thuillier, Elodie Nevado, Gaelle Maillan, Jeremy Jost, Sophie Leobon, Nicole Tubiana-Mathieu, Sandrine Lavau-Denes

**Affiliations:** 1 Department of Medical Oncology, University Hospital, Limoges, France; 2 Réseau Onco-Nouvelle-Aquitaine, Saint-Benoît, France; 3 Department of Pharmacy, University Hospital, Limoges, France

**Keywords:** Oral therapies, Cancer, Co-ordination, Multidisciplinary management

## Abstract

Oral therapies have highly modified cancer patient management and changed hospital practises. We introduce a specific Oral Therapy Centre and retrospectively review information prospectively recorded by co-ordination nurses (CNs) (the DICTO programme). We describe the roles played by CNs in the management of oral cancer therapies at Limoges Dupuytren Hospital between May 2015 and June 2018. All cancers, irrespective of stage or whether oral general chemotherapy or targeted therapy was prescribed, are included. We followed up 287 patients of median age 67 years (range 26–89 years). Of these, 76% had metastases and 44% were on first-line therapy. The vast majority (88%) of their first CN contacts occurred just after physician consultation and lasted an average of 60 min. As part of follow-up, the CNs made 2719 calls (average 10 min) to patients to educate them and to verify compliance and drug tolerance. They also received 833 calls from patients (70%) or their relatives or health professionals (30%) seeking advice on management of side effects. In addition to the initial appointments, 1069 non-scheduled follow-up visits were made to assess side effects (49.2%). The CNs devoted 5 h to each patient over 3 months of treatment (i.e. 25 min/day) and, also organised scheduled hospitalisations in the department of oncology for 51% of patients. We show the interest and real-life work in a specific oral therapy centre within oncology department with the role of CNs to facilitate the global health care of the patients.

**Electronic supplementary material:**

The online version of this article (10.1007/s12032-020-01393-7) contains supplementary material, which is available to authorized users.

## Introduction

Recent developments in oral chemotherapy and targeted therapies have radically changed cancer patient management; ambulatory care is now common. Oral therapies afford many advantages, improving patient quality of life and autonomy by reducing hospital stays and involving patients in their own care [1, 2]. During oral treatment, early detection of adverse events avoids emergency hospitalisation, and non-adherence to/discontinuation of treatment [3]. Although patients prefer oral to intravenous therapy [4, 5], non-adherence may become an issue because patients are not constantly supervised [6]. Multidisciplinary care is required to follow this therapy. Healthcare organisations must prioritise coordination to ensure the quality and safety of patient care [7, 8]. Optimising the care of cancer patients at home requires the co-ordinated intervention of hospital and town healthcare professionals to detect, prevent, and manage adverse events, avoid drug interactions, and educate patients. Several studies have revealed the benefits of managing patients treated on oral cancer drugs, both in terms of improved adherence and reduced toxicity. Usually, nurses are involved in patient contact, sometimes via telephone or other forms of communication [9–12].

The 2014–2019 cancer plan emphasises the need to re-organise care delivery, to strengthen town-hospital links, and to train medical and paramedical professionals [13]. Currently, no standard of care for cancer patients on oral therapy exists; few programmes treat a wide spectrum of cancers or involve all healthcare professionals [14]. To improve co-ordination of oral anti-cancer treatments, University Limoges Hospital developed a specific centre featuring strong links between physicians, nurses, and pharmacists of the hospital and town. In 2010, Limoges Hospital became 1 of 35 hospitals selected to develop a personalised approach to cancer treatment (the PICSCEL project) [15]. This highlighted the needs of certain patients in terms of care co-ordination between the town and hospital. In 2014, we were asked to develop a new centre to follow-up cancer patients on oral therapy and to link town and hospital healthcare professionals. The centre is an integral part of our Department of Medical Oncology. Here, we describe the centre’s operations with a focus on the roles played by co-ordinating nurses (CNs) (within the DICTO programme) who prospectively recorded their work and the tools used.

## Patients and methods

This was a single-centre prospective study of the roles played by CNs in the management of cancer patients on oral therapy prescribed by the Limoges Dupuytren Hospital. We included all cancer patients (metastatic or not) on oral chemotherapy and/or targeted therapy, at the request of the referring doctors, managed from May 2015 to June 2018.

### Objectives

We describe the work of the CNs. We explain their roles in detail, the contributions made, the added value of their care, and communications between hospital and non-hospital health professionals.

### Eligibility criteria

The oncologists proposed the DICTO programme to patients commencing oral therapy. The eligibility criteria were fluency in French, age 18 years or older, a diagnosis of a solid tumour requiring oral chemotherapy or targeted therapy, a willingness to be followed up, and a telephone at home. The exclusion criteria were hormonotherapy alone, no home telephone, inclusion in a clinical trial, a previous treatment with oral drugs, or a need for combined oral and intravenous treatments. Patients on multiple oral therapies were included. Patients were censored on death, disease progression requiring initiation of intravenous treatment, or on request.

### Organisation of the oral therapy centre (OTC)

The OTC is a dedicated unit in the Department of Medical Oncology, with a consultation room and a nursing office. A dedicated hotline facilitates communication between CNs, patients, their family members, and other healthcare professionals. The hotline is open from Monday to Friday from 10 am to 6 pm. The OTC includes oncologist practitioners, CNs trained in the management of oral treatments, health professionals offering supportive care, pharmacists, and a social worker. The six CNs have been trained in oral therapy oncology management. One or two CNs are assigned to OTC daily to follow-up patients. The CNs also arrange consultations with doctors when necessary. When patients visit, pharmacy consultations are also offered to adapt treatments (if necessary), monitor side effects, and explore patient attitudes.

#### Procedure at initial consultation

After the patient consulted with the oncologist and oral treatment was prescribed, the CNs assumed responsibility for patient care. Twenty-four oral chemotherapies and targeted therapies were delivered (Supplementary Table 1). For each drug, the CNs had been trained, and had information sheets on how to deal with: biology (essential blood tests), telephone intervals (and modifications thereof if patients visited for blood tests), medical consult intervals, monitoring of specific toxicities, and when to call the referring doctor.

The CNs had been trained to deal with each type of medication, had information sheets on how to take essential blood tests into account, establish the intervals for medical consultations, monitor specific toxicities and determine when to call the referring doctors.

The CNs explained to the patients (and their families, if possible) drug indications, dosages, administration, adverse reactions, and drug interactions. During initial appointments, CNs gave patients drug data and follow-up notebooks, finalised personalised care plans, assessed social precariousness and obtained G8 scores [16], contacted social services if social fragility was evident, ensured that the secretariat sent letters and drug data to general practitioners, explained the prescriptions, called home nurses and town pharmacists and sent all necessary documents, and programmed medical consults.

We identified high-risk situations at initial consultation and special attention (extra telephone calls and systematic social and psychological support) was given to these patients. It included patients with brain tumours, pancreatic cancers, or head-and-neck cancers (because of poor prognoses or a high risk for a complex short- or long-term disease course); with metastases or inoperable local recurrences; over 75 years of age; and at risk for social fragility as revealed by the INCA indicators [17]. The INCA social fragility indicators are over 75 years of age, living alone, disability, difficulty speaking French, difficult financial situation, and difficulty performing daily activities. A patient might exhibit several risk factors.

#### Follow-up

All patients were followed up throughout their treatments via telephone and consultations. Follow-up was individualised by the type of drug and patient characteristics (based on questionnaire responses) and formalised in written or computerised notes held in the OTC. All questionnaires were developed and validated by a working group composed of doctors, nurses, and pharmacists [18]. Phone calls were made on days 15 and 30, and then every 3 months. An additional call for high-risk patients was made between day 8 and 10. During each call, the CN assessed compliance and drug tolerance, educated the patient, answered questions, reinforced the initial advice, and planned the next visit or telephone call.

CNs contacted local nurses, general practitioners, local pharmacists, or paramedical staff, depending on the patient’s situation. Hospitalisation was discussed with the oncologists who prescribed the drugs. If hospitalisation was necessary, the CNs recorded why and the outcomes of treatment. The CNs actively facilitated the patient’s return home by calling all town caregivers. All direct or indirect actions were recorded on a computer spreadsheet.

Clinical data were collected in accordance with French bioethics laws addressing patient information and consent. This project has been ethically approved by the relevant national authority: the National Institute of Cancer (INCa) and the Ministry of Health (no. DGOS/R3/2014/235–24 July 2014). Patients provided written (signed) informed consent to use their medical and administrative data.

#### Statistical analyses

All data were collected and analysed using STATVIEW software (SAS Institute Inc., Cary, NC, USA). Quantitative data are given as medians ± standard deviations (SDs) and qualitative results are numbers with percentages. The significance of between-group differences was evaluated using the Chi-square test. Means were compared using the non-parametric Mann–Whitney *U*-test for continuous variables and the Wilcoxon signed-rank test for categorical variables. A *p*-value < 0.05 was taken to reflect significance.

## Results

### Patient population

**Fig. 1:**
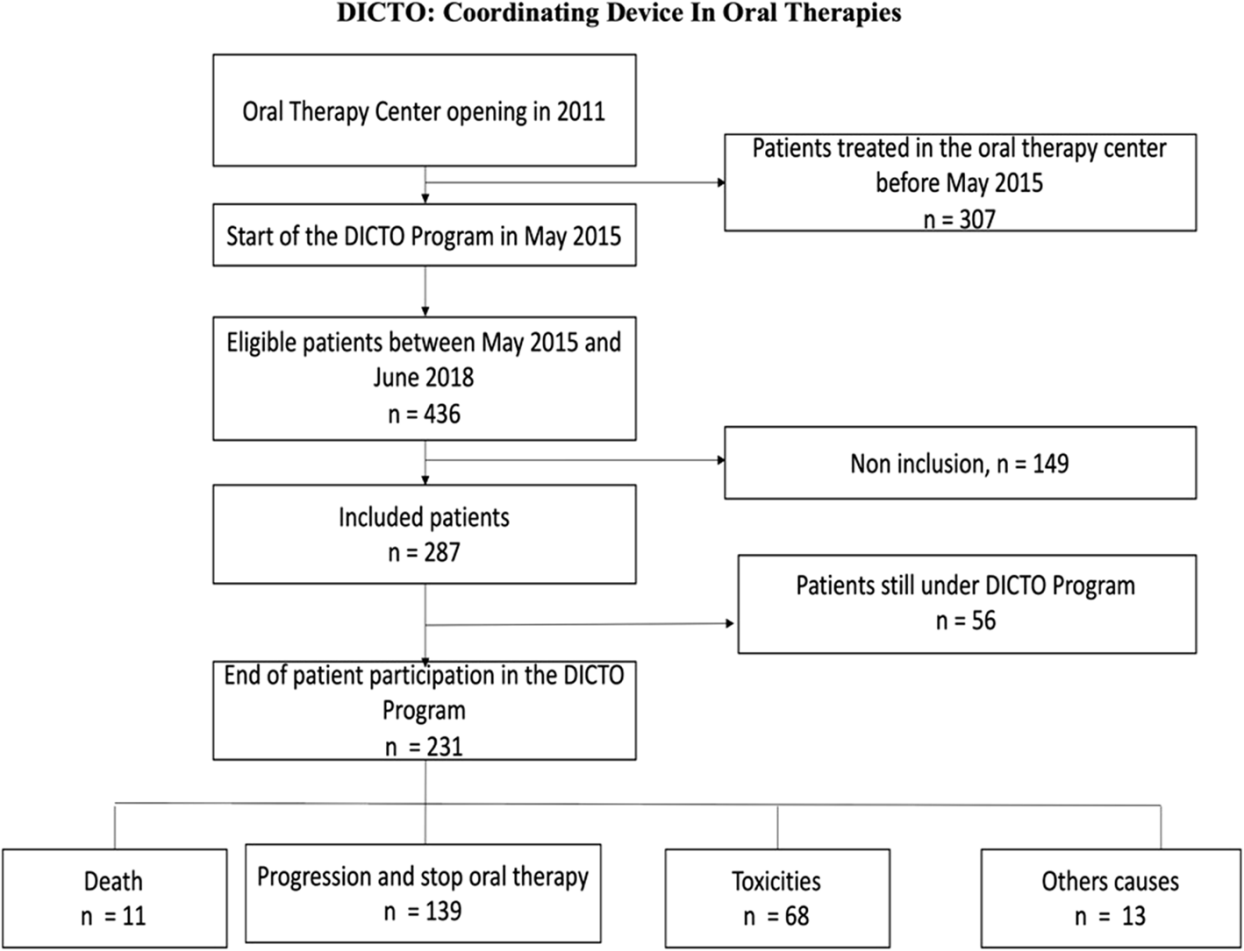
Flow chart of the DICTO programme

Of 436 patients on oral treatments, 287 were recruited between May 2015 and June 2018 (Fig. 1). Demographic characteristics are shown in Table 1. We included 155 females and 132 males of median age 67 years (range 26–89 years); 63 were older than 75 years (Supplementary Fig. 1). Most patients lived in Limousin (Haute-Vienne, Creuse, Corrèze) (74%); the others lived in neighbouring areas or sometimes in more distant places (Supplementary Fig. 2). The sites of primary cancer varied. The most common cancers were breast (30%), renal (19%), colorectal (12%), and brain cancers (10%) (Table 1). Most cancers were metastatic (76%); 67 patients were prescribed neoadjuvants. Most patients (131) were considered high-risk because of metastases. Other high-risk INCA indicators included age > 75 years (*n* = 63), social disadvantage (*n* = 36), and tumour type (*n* = 32) (these sometimes overlapped).

**Table 1:** Tumour and patient characteristics

	*N* (%)
Number of patients	287
Sex	
Male	132 (46)
Female	155 (54)
Median age (minimum to maximum) (years)	67 (26–89)
Patients aged > 75 years	63 (22)
Median G8 score (29 cases) (minimum to maximum)	12 (6–16)
High-risk patients as revealed by INCA	131 (45.6)
Primary tumours	
Breast cancer	88 (30)
Renal cancer	54 (19)
Colorectal cancer	34 (12)
Brain tumour	30 (10)
Prostate cancer	19 (7)
Sarcoma	13 (4.5)
Lung cancer	11 (4)
Neuro-endocrine pancreatic cancer	11 (4)
Hepatocellular carcinoma	8 (3)
Ovarian cancer	7 (2.5)
GIST	4 (1.4)
ACUP	3 (1)
Thyroid cancer	3 (1)
Cholangiocarcinoma	2 (0.6)
Time between initial diagnosis and study inclusion (months); median (minimum to maximum)	23 (1–286)
Treatment characteristics	
Neoadjuvant or adjuvant setting	67 (24)
Metastatic setting	220 (76)
Therapy line in metastatic setting (median, minimum to maximum)	2 (1–9)

Data are *n* (%) unless indicated otherwise

High risks included brain and pancreatic cancers, metastatic cancer, inoperable local recurrence, social fragility as revealed by INCA, and age over 75 years

### Treatment

The median time between the first diagnosis of cancer and oral therapy commencement was 23 months (1–286 months). Of patients with metastases, 98 (44%) were on first-line, 51 (24%) on second-line, and 71 (32%) on third- or later-line drugs (Table 1). Supplementary Table 1 lists the treatments; 68% were targeted (*n* = 15). Everolimus was most commonly prescribed (17.4%), followed by capecitabine (13.5%) and palbociclib (9.4%). Sunitinib and everolimus induced the most side effects (39% and 38%, respectively) followed by regorafenib (36%) and pazopanib (36%). Most participants developed disease progression requiring new intravenous treatment (48.4%), side effects (24%), or died (4%). No patients withdrew from the programme. The median treatment duration was 4 months (range 0–37 months) (Supplementary Table 1).

### Management of patients by CNs

The first CN contact lasted for an average of 60 min (20–120 min) and usually occurred just after the patient visited the doctor (*n* = 255, 88%). The consultations were longer for patients older than 75 years (60 vs. 50 min, *p* = 0.01, respectively). Consultation length did not vary by other high-risk factors or the distance between the hospital and the patient’s home. Information sheets were given to all patients (except two who refused them). If a follow-up booklet was available, it was given to the patients (254; 88%). Personalised care was scheduled for 253 patients (88%) (Table 2). A questionnaire exploring satisfaction was completed by the first 30 patients; feedback was positive.

**Table 2:** Actions taken during initial appointments

	*N* (%)
Total	287
Contact with general practitioners:	
Contact made by telephone: yes/no	254 (88)/32 (11)
Absence of contact with a general practitioner because …	
General practitioner not available (answering machine or secretary)	13 (40)
Other reasons (unknown phone number, lack of time)	13 (40)
Absence of a patient-designated doctor	6 (18)
Duration of calls to the general practitioner (min)	10 (3–43)
Letters sent to general practitioners: yes/no	234 (81)/47 (16)
Information sent to general practitioners: yes/no	225 (78)/52 (18)
Contact with town nurses:	
Contact by telephone: yes/no	188 (65)/98 (34)
Absence of contact with town nurses because…	
Absence of patient-designated nurse	70 (72)
Other reasons (unknown phone number, lack of time)	25 (25)
Nurse not available	3 (3)
Duration of calls to town nurse (min)	6 (2–30)
Contact with town pharmacists	
Contact by telephone: yes/no	242 (84)/44 (15)
Absence of contact with town pharmacists because…	
Medication given by hospital	26 (60)
Other reasons (unknown phone number, lack of time)	8 (18)
Absence of a patient-designated pharmacist	7 (16)
Pharmacist not available	3 (6)
Duration of call to pharmacist (min)	10 (2–40)
Total length of CN calls (pharmacist + general practitioner + town nurse)	20 (2–65)
Contact with social services	46
Duration of call to social services (min)	30 (5–185)
Other calls	72
Requests for additional examinations	51 (71)
Other	9 (12.5)
Hospital nurses	5 (7)
Hospital doctors	4 (5.5)
Town laboratory	2 (3)
Dieticians	1 (1)
Duration of calls (min)	10 (5–20)

During follow-up, of the 2720 calls made by CNs, 1869 were to patients (66.3%) and 226 were to helpers and family members. Calls dealt with test results (926/1345; 68.8%), systematic follow-up (658/2719; 24.2%), and treatment validation (434/2719; 16.0%) (Table 3). CNs received 833 calls of median duration 10 min (2–135 min) from patients; 70% were calling from places distant from family members (Table 4). The calls explored management of side effects (226/334) and test data validating drug prescription (*n* = 84). Some patients complained of anxiety/a need for moral support (*n* = 71). Other calls dealt with misunderstandings/reformulations of plans (49/79; 62%) (Table 4). CN consultations in the OCT (median duration 30 min) were scheduled on 1,069 occasions. Of the 287 patients, 238 received a mean of 3 such consultations (range 1–18). Side effects were assessed during either non-scheduled (49.2%) or scheduled (50.8%) appointments (Table 5). Appointment numbers did not vary by patient geographic location or high-risk status. The CNs also provided follow-up care for patients after 248 hospitalisations (a median of 2 appointments per hospitalised patient [range 2–12 hospital stays]). Of all hospitalisations, 125 were in the department of oncology (scheduled, 51%), 81 were in the emergency department, and 23 were in other departments (unscheduled, 49%).

**Table 3:** Planned and unplanned calls made by oral therapy centre nurses during follow-up

	Total
Number of planned calls^a^	2719
Number of calls per patient made during follow-up; median (minimum to maximum)	15 (1–58)
Duration of calls (min); median (minimum to maximum)	10 (2–130)
Persons called, *n* (%)	2821
Patients	1869 (66.3)
Town laboratory	469 (16.6)
Patient helpers and families	226 (8.0)
Hospital services	60 (2.5)
Pharmacists	52 (1.8)
Persons responsible for additional examinations	39 (1.3)
General practitioners	27 (1.0)
Town nurses	34 (1.2)
Nurses in the medical oncology department	20 (0.7)
Others	14 (0.5)
Social services	7 (0.2)
Psychologists	4 (0.1)
Causes of calls, *n* (%)	2719
Test results	1345 (49.4)
Systematic follow-up	658 (24.2)
Treatment validations	434 (16.0)
Administrative information	168 (6.2)
Medical information	74 (2.7)
Concomitant treatments	25 (1.0)
Education	15 (0.5)
Actions taken after phone calls, *n* (%): unplanned calls	507
Contact hospital doctors	308 (60.7)
Request prescriptions	81 (16)
Contact general practitioners	27 (5.3)
Requests for home test check-ups	25 (4.9)
Emergency hospitalisation	17 (3.3)
Contact town nurses	11 (2.2)
Reformulated hygiene/dietary advice	11 (2.2)
Contact psychologists	9 (1.8)
Contact patients	8 (1.6)
Direct hospitalisation in a ward	6 (1.2)
Contact nurse co-ordinators	2 (0.4)
Contact family/caregiver	1 (0.2)
Contact social services	1 (0.2)

^a^Planned calls were made on days 8–10 for high-risk patients, then on days 15 and 30 and every 3 months thereafter

**Table 4:** Unplanned calls received and made by oral therapy centre nurses during follow-up

	Total
Number of calls received	833
Number of calls per patient during follow-up; median (minimum to maximum)	6 (1–25)
Duration of calls received (min); median (minimum to maximum)	10 (2–135)
Persons initiating calls, *n* (%)	833
Patients	586 (70)
Patient helpers	93 (12)
Family members	58 (7)
Hospital services (other)	24 (3)
Town nurses	26 (3)
General practitioners	19 (2)
Pharmacists	12 (1.3)
Nurses in the medical oncology department	8 (0.9)
Town laboratory	6 (0.7)
Hospital service (additional examinations)	1 (0.1)
Causes of calls, *n* (%)	884
Side effects	334 (37.8)
Test results received	84 (9.5)
Misunderstanding/reformulation	79 (9.0)
Request for prescription	74 (8.4)
Anxiety/moral support	71 (8.0)
Administrative information	59 (6.7)
Appointment confirmation	47 (5.3)
Other	46 (5.2)
Changing an appointment	37 (4.1)
Concomitant treatments	32 (3.6)
Request hospitalisation (emergencies)	10 (1.1)
Request new appointments	6 (0.7)
Hygiene/dietary advice	5 (0.6)
Actions taken after calls, *n* (%)	507
Contact hospital doctors	308 (60.7)
Requests for prescriptions	81 (16)
Contact general practitioners	27 (5.3)
Requests for home test check-up	25 (4.9)
Emergency hospitalisation	17 (3.3)
Contact town nurses	11 (2.2)
Reformulated hygiene-dietary advice	11 (2.2)
Contact psychologists	9 (1.8)
Patient contact	8 (1.6)
Direct hospitalisation in a ward	6 (1.2)
Contact nurse co-ordinators	2 (0.4)
Contact family/caregiver	1 (0.2)
Contact social services	1 (0.2)

**Table 5:** Appointments in the oral therapy centre

	Total
Number of appointments	1069
Number of appointments per patient; median (minimum to maximum)	6 (1–18)
Duration of appointment (min);median (minimum to maximum)	30 (10–410)
Reasons, *n* (%)	
Side effects	526 (49.2)
Scheduled appointments	543 (50.8)
After-appointment contacts, *n* (%)	78
Hospital doctors	55 (70.5)
Town nurses	9 (11.5)
General practitioners	5 (6.5)
Psychologists	5 (6.5)
Social services	2 (2.6)
Family/caregivers	1 (1.2)
Co-ordinating nurse	1 (1.2)
Actions taken after appointments, *n* (%)	103
Requests for home test check-ups	50 (48.6)
Direct hospitalisation in a ward	19 (18.4)
Requests for prescriptions	18 (17.4)
Reformulated hygiene/dietary advice	16 (15.6)

### Town-hospital links created by CNs

At the first consultation, the CNs telephoned the patient’s general practitioner (88%), town nurse (65%), and town pharmacist (84%). The combined median duration of all calls was 20 min (range 2–65 min). Letters and booklets were sent to general practitioners (Table 2). Of patients at high INCA-defined risk, 46 were referred to social services during initial consultations; some refused or claimed that assistance was already in place. The CNs also contacted hospital nurses, dieticians, psychologists, town laboratories, and hospital doctors and, also requested additional examinations (*n* = 72) (Table 2).

During follow-up, CNs made threefold more calls than they received (2719 [median time 10 min; range 2–130 min] vs. 833). A total of 726 calls were to health professionals (Table 3) to discuss test results (419/1345 tests; 31%) and administrative data (67/168 datasets; 40%), and to seek medical information (44/74 questions; 60%). CNs made unplanned calls to doctors if decisions lay outside CN competence (60%) and to order prescriptions (16%) (Table 3). The number of calls did not vary by patient geographic home or risk status (low or high). Of the 833 unplanned calls received by CNs, 96 were from concerned health professionals who discussed toxicities (32/334; 9.5%), misunderstandings/reformulations (19/79; 24%), and administrative details (16/59; 27%) (Table 4).

In conclusion, overall, the CNs devoted 5 h to each patient over 3 months, including calls and consultations. A total of 1847 calls were unplanned, related to patient requests or actions required following planned calls.

## Discussion

The use of oral therapies in oncology has increased over the past decade, necessitating changes in the monitoring of side effects; this is essential to ensure patient compliance, assess safety, and optimise therapy. Here, we describe the pivotal roles played by the CNs of a multidisciplinary OTC team. The CNs optimised communications between patients and healthcare professionals. The CNs of the OTC involved outside healthcare professionals in patient care irrespective of cancer or drug type. To the best of our knowledge, few organisations have used CNs, and hospital and town pharmacists and doctors, to manage oral therapies for all types of cancer patients [19]. Interventions included education, monitoring, administration, telephone calls, and physical assessments; high-risk patients often do not receive so much attention [19]. No patients withdrew and the rate of side effects was low (24%); the OTC worked well. Full-time CNs are required to adequately co-ordinate town and hospital healthcare professionals. The call register showed that not only patients, but also their families, needed to contact the OTC. One or two nurses were always available to take calls and then to alert the necessary professionals. Certain drugs associated with serious side effects (e.g. everolimus) were specifically monitored [20] reducing treatment discontinuation [21]. When published, the outcomes of CAPRI, a rare randomised controlled trial enrolling breast cancer patients, are expected to confirm the critical roles played by nurse “navigators” and a Web portal in terms of co-ordinating town and hospital carers during routine delivery of oral anti-cancer therapy [12].

Although patients prefer oral drugs, and although out-patient chemotherapy improves the quality of life [22], management of anxiety experienced by patients and their families remains a major issue [2, 23, 24]. Some anxiety is attributable to side effects; patients on oral therapy are not monitored to the same extent as those on intravenous therapy [2, 23, 24].We found that 37.8% of calls received concerned side effects (the prime reason for calling). It is essential to manage treatment, improve patient quality of life, and provide psychological assistance. Whether via the telephone or otherwise, patients must have information [25, 26].

A co-ordinated regional health network of the Ile de France (CHIMORAL) has reported findings [14]. The hospitalisation rates of orally and intravenously treated cancer patients did not differ. Patients reporting satisfaction with treatment and minimal anxiety adhered well to oral chemotherapy [1]. We thus evaluated patient quality of life. The CNs reduced emergency admissions and physician visits, and thus were cost-effective, although further prospective studies are required [27]. A novel feature of our work is that we included pharmacists when co-ordinating patient management. A prospective study involving 18 French cancer centres found that only 54.5% had deployed oral anti-cancer drugs programmes that involved pharmacists, and only 44.4% featured hospital/community co-ordination [28]. Community pharmacists educate patients but also require cancer-specific education [29, 30]. Calls from nurses to general practitioners are very well accepted if the nurses are trained in oral cancer treatment. A possible limitation of our work is that we included all types of cancers and treatments, but we deliberately chose to analyse the entire population, because in the real world, our oncology department is responsible for a very large area. Another limitation is that we sought feedback from only the first 30 patients, and we did not survey hospital and town healthcare professionals.

In conclusion, we developed and implemented a new healthcare structure for cancer patients on oral therapy; all patients received personalised support throughout their treatment. Our OTC co-ordinated all hospital and town professionals who “surrounded” the patient. The OTC improves patient quality of life, reduces anxiety, and improves compliance.

## Electronic supplementary material

Below is the link to the electronic supplementary material.

Supplementary file1 (DOCX 199 kb)

## Data Availability

The datasets used and/or analysed in the current study are available from the corresponding author upon reasonable request.
