# Supplementary material for: Prospective evaluation of an anti-cancer drugs management programme in a dedicated oral therapy center (DICTO programme)
Source: Med Oncol. 2020 Jul 25;37(8):69. doi: 10.1007/s12032-020-01393-7 (PMC7382654; doi:10.1007/s12032-020-01393-7)
Supplement: Supplementary file 1 — Supplementary file1 (DOCX 199 kb) [file 12032_2020_1393_MOESM1_ESM.docx]

**Supplementary table 1 Treatments and reasons of their stopped**

| Treatments | Number | Progression | Side effects | Death during the follow-up | Treatments were not discontinued | Others causes |
| --- | --- | --- | --- | --- | --- | --- |
| Everolimus/Afinitor® | 59 | 27 | 21 | 1 | 5 | 5 |
| Cabozantinib/Cabometyx® | 7 | 2 | 2 | 0 | 3 | 0 |
| Vandetanib/Caprelsa® | 2 | 1 | 1 | 0 | 0 | 0 |
| Etoposide/ Celltop® | 2 | 2 | 0 | 0 | 0 | 0 |
| Cyclophosphamide/Endoxan® | 4 | 4 | 0 | 0 | 0 | 0 |
| Afatinib/Giotrif® | 2 | 2 | 0 | 0 | 0 | 0 |
| Imatinib/Glivec® | 5 | 1 | 0 | 0 | 3 | 1 |
| Palbociclib/Ibrance® | 32 | 15 | 0 | 0 | 17 | 0 |
| Axitinib Inlyta® | 4 | 4 | 0 | 0 | 0 | 0 |
| Trifluridine/Tipiracil/ Lonsurf® | 8 | 3 | 0 | 3 | 2 | 0 |
| Olaparib/Lynparsa® | 2 | 2 | 0 | 0 | 0 | 0 |
| Navelibine/ Navelbine® | 26 | 21 | 3 | 0 | 1 | 1 |
| Sorafenib/Nexavar® | 11 | 8 | 3 | 0 | 0 | 0 |
| Regorafenib/Stivarga® | 15 | 8 | 4 | 1 | 2 | 0 |
| Sunitinib/Sutent® | 30 | 16 | 9 | 0 | 0 | 5 |
| Erlotinib/Tarceva® | 3 | 3 | 0 | 0 | 0 | 0 |
| Temodal/Temodal® | 32 | 21 | 1 | 6 | 4 | 0 |
| Lapatinib/Tyverb® | 2 | 1 | 0 | 0 | 1 | 0 |
| Pazopanib/Votrient® | 28 | 16 | 8 | 0 | 4 | 0 |
| Crizotinib/Xalkori® | 1 | 1 | 0 | 0 | 0 | 0 |
| Capecitabine/Xeloda® | 46 | 26 | 13 | 0 | 7 | 0 |
| Enzalutamide/Xtandi® | 3 | 1 | 1 | 0 | 0 | 1 |
| Abiraterone/Zytiga® | 16 | 7 | 2 | 0 | 7 | 0 |
| Total | 340 | 192 | 68 | 11 | 56 | 13 |

Targeted therapies are grayed out. Death during the follow-up was not related to toxicites. Others included 7 changes of therapy center by the patient and 6 therapeutic breaks.

**Supplementary Figure 1 : Age distribution of patients**

**Supplementary Figure 2 Geographic origin of patients**

Number (n) of patients in each aeras ; 213 were considered as “near” from Limoges hospital (departments 87, 19 and 23) and 74 patients were considered as “distant” from Limoges hospital
